# Production of Cytotoxic Antibodies After Intra-Articular Injection of Allogeneic Synovial Membrane Mesenchymal Stem Cells With and Without LPS Administration

**DOI:** 10.3389/fimmu.2022.871216

**Published:** 2022-04-27

**Authors:** Gustavo dos Santos Rosa, André Massahiro Teramoto Krieck, Enrico Topan Padula, Fernanda de Castro Stievani, Mariana Correa Rossi, João Pedro Hübbe Pfeifer, Roberta Martins Basso, Aline Márcia Marques Braz, Márjorie de Assis Golim, Ana Liz Garcia Alves

**Affiliations:** ^1^ Department of Veterinary Surgery and Animal Reproduction, Regenerative Medicine Lab, School of Veterinary Medicine and Animal Science, São Paulo State University (UNESP), Botucatu, Brazil; ^2^ Department of Veterinary Clinics, School of Veterinary Medicine and Animal Science, São Paulo State University (UNESP), Botucatu, Brazil; ^3^ Flow Cytometry Laboratory, Applied Biotechnology Laboratory, Clinical Hospital of Botucatu Medical School, Botucatu, Brazil; ^4^ Graduate Program in Research and Development (Medical Biotechnology), Botucatu Medical School, São Paulo State University (UNESP), Botucatu, Brazil

**Keywords:** humoral immune response, mesenchymal stromal cells, MHC, intraarticular injection, microcytotoxicity, cell rejection reactions, horses

## Abstract

Allogeneic mesenchymal stem cells (MSC) are widely used in clinical routine due to the shorter expansion time and reliability of its quality. However, some recipients can produce alloantibodies that recognize MSCs and activate the immune system, resulting in cell death. Although antibody production was already described after MSC injection, no previous studies described the immune response after intra-articular MSC injection in acute synovitis. This study aimed to evaluate the influence of inflammation on immune response after single and repeated intra-articular injections of synovial membrane MSC (SMMSC). Horses were divided in three groups: control group (AUTO) received autologous synovial membrane MSCs; whereas group two (ALLO) received allogeneic SMMSCs and group three (ALLO LPS) was submitted to acute experimental synovitis 8 h before SMMSCs injection. The procedure was repeated for all groups for 28 days. Physical and lameness evaluations and synovial fluid analysis were performed. Sera from all animals were obtained before and every 7 days after each injection up to 4 weeks, to perform microcytotoxicity assays incubating donor SMMSCs with recipients’ sera. The first injection caused a mild and transient synovitis in all groups, becoming more evident and longer in ALLO and ALLO LPS groups after the second injection. Microcytotoxicity assays revealed significant antibody production as soon as 7 days after SMMSC injection in ALLO and ALLO LPS groups, and cytotoxicity scores of both groups showed no differences at any time point, being equally different from AUTO group. Although inflammation is capable of inducing MHC expression in MSCs, which enhances immune recognition, cytotoxicity scores were equally high in ALLO and ALLO LPS groups, making it difficult to determine the potentiation effect of inflammation on antibody production. Our findings suggest that inflammation does not display a pivotal role in immune recognition on first allogeneic MSC injection. In a translational way, since specific antibodies were produced against MSCs, patients that need more than one MSC injection may benefit from a first allogeneic injection followed by subsequent autologous injections.

## 1 Introduction

Current treatments for joint injuries aim to reduce pain, inflammation, and articular degradation. Cell-based therapy appeared as a new alternative capable of providing better results in these cases (1). In this sense, mesenchymal stem cells (MSCs) have been used due to their trophic, immunomodulatory, and differentiation properties (2, 3).

Studies evaluating biosafety of allogeneic MSCs injections as an alternative to autologous injections have been published (4–8). Isolated and repeated injections of allogeneic MSCs in horses under several conditions (9–11) can generate positive results in different injuries and administration routes (12–14). However, little data is known regarding the humoral reaction after repeated intra-articular injections of allogeneic MSCs in already established acute inflammatory conditions (15).

The major histocompatibility complex (MHC) is a group of cell surface glycoproteins capable of initiating specific immune responses (16). While MSCs usually express MHC-I, the expression of MHC-II is heterogeneous (6, 11, 17) and influenced by some factors like passage number and presence of interferon-gamma (IFN-γ). Likewise, pro-inflammatory cytokines such as IFN-γ and tumor necrosis factor alpha (TNF-α) are released in tissue inflammation, which also activates the innate immune system (18, 19).

Specific alloantibodies against donor MSCs can be produced after allogeneic MSCs injections in horses, even when MHC-II negative MSCs are used (5, 7). Thus, the evaluation of allogeneic MSCs behavior in inflammatory environments is extremely important, once MHC-II negative MSCs have shown MHC-II expression after being exposed to IFN-γ *in vitro (* 6) which could enhance MSCs immunogenicity and impair subsequential allogeneic injections.

Synovial membrane is a relatively novel MSC source, and synovial membrane-derived mesenchymal stem cells (_SM_MSC) have demonstrated superior chondrogenic potential (20) and notable potential for articular repair (21). Additionally, similarities between human and equine species place the last as good experimental models for translational orthopedic research of naturally occurring musculoskeletal lesions (22–24).

This study compared clinical and immune reactions before and after repeated injections of allogeneic _SM_MSC in healthy and inflamed joints, aiming to elucidate the influence of inflammation on immune recognition, inflammatory events, and alloantibody production.

## 2 Material and Methods

### 2.1 Ethics Committee Approval

This study was performed according to the ethics committee of São Paulo State University (protocol n. 0240/17).

### 2.2 Animals

Fifteen naïve mixed breed geldings were used in this study, with mean age of 5 years and mean weight of 340 kg. There were no blood relationships that could indicate partial or total matching among them. Exclusion criteria were any alterations in physical exams or lameness scores, presence of synovial effusion and radiographic or ultrasonographic abnormal findings. Horses with previous blood transfusions, MSC injections, or any kind of allogeneic grafts were also not included in the study.

### 2.3 Study Design

Allogeneic synovial membrane MSCs from three healthy donors were injected in 15 tibiotarsal joints, divided in three groups. One group was composed of healthy joints (ALLO group, n=6). The second group (ALLO LPS, n=6) was submitted to experimental acute synovitis 8 h before _SM_MSC injection. Synovitis was induced using purified LPS (0.5 ng in 2mL of PBS). Autologous injection of _SM_MSC was performed on the three donor horses as negative control (AUTO group, n=3).

Injections of _SM_MSC from the donor randomly selected were performed at the initial time point and repeated after 4 weeks. Synovial fluid (SF) samples were collected at time points 0 h, 24 h, 72 h and 7, 14, 21, and 28 days after each injection (i.e., T0, T1, T3, T7, T14, T21, and T28 for the first injection, and T28, T29, T31, T35, T42 T49, and T56 for the second injection, respectively). Blood sera were collected before injections and every 7 days for 8 weeks to perform microcytotoxicity assays. Haplotyping was not performed in this study.

### 2.4 Isolation, Culture, and Characterization of _SM_MSC

Synovial membrane was harvested from the radiocarpal joint by arthroscopy. The sample was washed in Knockout DMEM (Gibco, Grand Island, NY, USA) and dissociated with type I collagenase (4 mg/g of sample) for 3 hours at 37°C and 5% of CO_2_. Enzymatic dissociation was stopped using DMEM with 10% of Fetal Bovine Serum (FBS - Gibco, Grand Island, NY, USA).

Sample was centrifuged at 720 x g for 10 minutes and the pellet was seeded in culture bottles of 75 cm^2^ (TPP-Techno Plastic Products, Zollstrasse, Trasadigen, Swiss) containing DMEM F12 Glutamax (Thermo Fisher Scientific, Grand Island, New York, USA) and 10% FBS, 1% antibiotic-antimycotic solution (10,000 units/mL of penicillin, 10,000 μg/mL of streptomycin, 25 μg/mL of Amphotericin B) (antibiotic-antimycotic - Thermo Fisher Scientific, Grand Island, New York, USA). After reaching 80% of confluence cells were detached using Trypsin-EDTA (0.25%) (Thermo Fisher Scientific, Grand Island, New York, USA) and transferred to 175 cm^2^ culture bottles. The procedure was repeated until cells reached fourth passage (P4).

**Figure 1:**
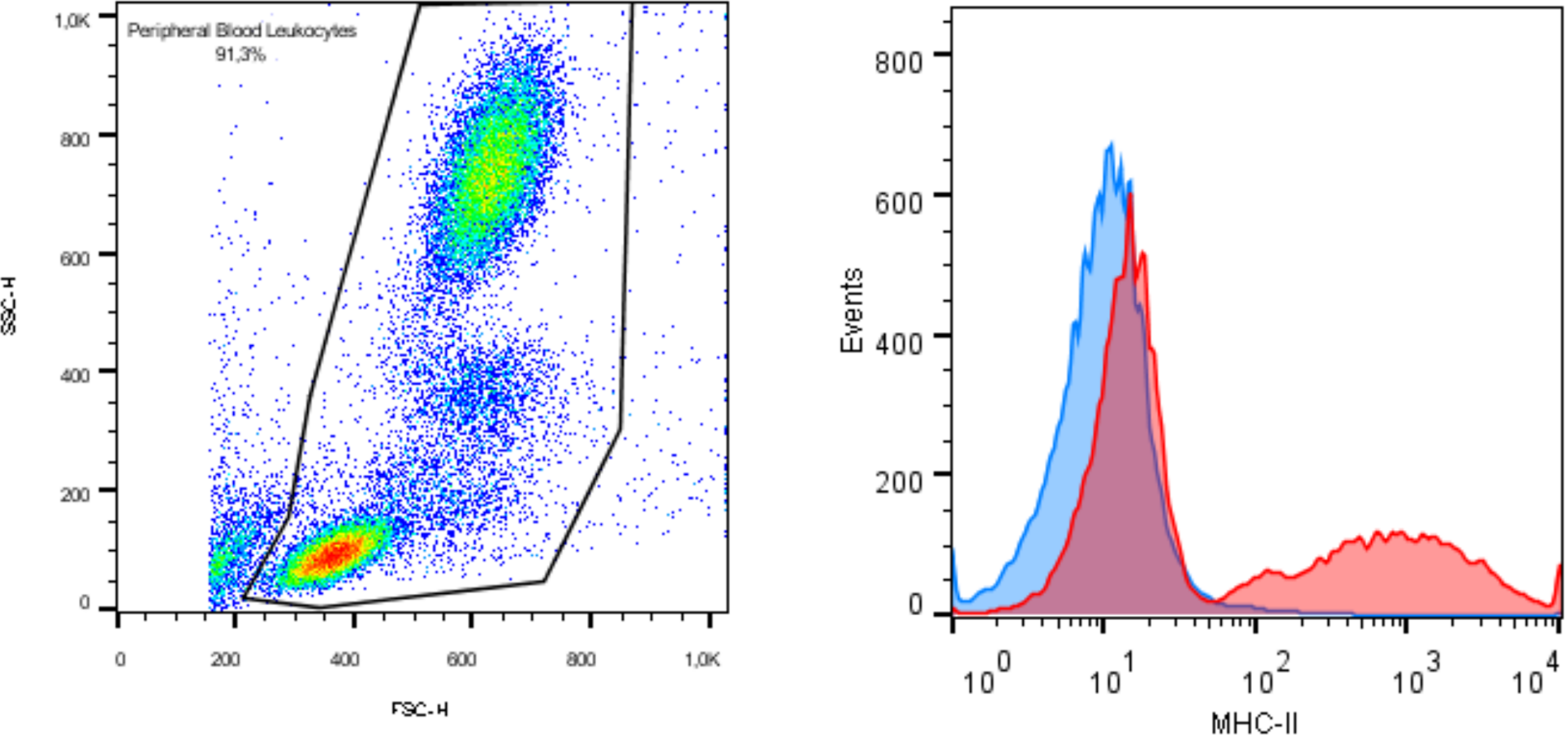
Immunophenotyping by flow cytometry exhibiting expression of MHC class II in equine peripheral blood as positive control.

Characterization was performed by flow cytometry evaluation of markers CD90, CD44, CD73, CD45, and MHC class II. Each monoclonal antibody was tested using appropriate equine cells as positive controls, as **Figure 1** illustrates positive expression of MHC-II in equine peripheral blood leukocytes. Trilineage differentiation assays were performed using commercial differentiation media (StemPro™ Osteogenesis, Adipogenesis and Chondrogenesis Differentiation Kits, Gibco, Grand Island, NY, EUA). Osteogenic and adipogenic differentiation were evaluated in adhered cell cultures exposed to the differentiation media for 14 days and stained with Alizarin Red S and Oil Red O, respectively. Chondrogenic differentiation was performed by exposing tridimensional cultures to the chondrogenic medium for 21 days. The pellet was histologically processed and stained with Alcian Blue to highlight extracellular matrix deposition.

Culture medium was replaced by DMEM F12 Glutamax without FBS 48 h before injection to remove the FBS component from the culture. Since DMEM with 10% FBS was used to stop trypsin activity on the day of the injection, cells were washed in PBS (Thermo Fisher Scientific, Grand Island, New York, USA) three times to deplete as much FBS as possible.

### 2.5 Induction of *Acute Synovitis* and _SM_MSC Injection

Tibiotarsal acute synovitis was induced in the ALLO group (n=6) 8 h before _SM_MSC injection, by intra-articular injection of 0.5 ng of Lipopolysaccharide from Escherichia coli serotype 055:B5 (Sigma-Aldrich^®^. Saint Louis, Missouri, USA) diluted in 2 mL (0.25 ng/mL solution) of PBS, according to Williams (25), which would be sufficient to cause a mild self-limiting synovitis for 7 days. Synovial fluid was sampled immediately before LPS injection. The procedure was repeated on the same animals after 4 weeks, before the second _SM_MSC injection.

Intra-articular _SM_MSC injection was performed using a 21G needle placed on the dorsomedial aspect of the tibiotarsal joint, either medial or lateral to the saphenous vein. Approximately 2 ml of synovial fluid were collected before the injection. Samples were transferred to EDTA (Ethylenediamine Tetraacetic Acid) tubes for cytological analysis. After arthrocentesis, joints received 10 (7) _SM_MSC diluted in 2 mL of PBS. Four weeks after the first injection the procedure was repeated in all groups.

Blood samples were collected from all animals every 7 days for 4 weeks following each _SM_MSC injection. Blood was centrifuged at 800 x g for 10 minutes at 4°C for serum collection. Serum samples were frozen at -80°C to determine antibody titration in microcytotoxicity assays.

### 2.6 Clinical Evaluation

Physical and lameness evaluations were performed at all time points to assess vital parameters and AAEP lameness scores. Joints were submitted to ultrasonographic evaluation using the ultrasound LOGIQ-e (GE Healthcare™, USA) with 10 MHz linear probe, to evaluate synovial fluid echogenicity, synovial membrane proliferation and synovial effusion. Synovial effusion was measured by the distance between talus and the joint capsule.

### 2.7 Synovial Fluid Analysis and Microcytotoxicity Assays

Synovial fluid analysis consisted of a macroscopic evaluation initially (aspect) and specific gravity, chemical examination through a quantitative analysis (urine sticks) for pH, protein, glucose, and blood. The total nucleated cell count/μL (TNCC) was performed in Neubauer’s chamber and the preparation of cytological slides was made using cytocentrifuge, centrifugation, or direct squash, according to the count. Cells were stained with Diff-Quick and the differential cell count was performed on a 1000x-oil objective.

Standard two-stage microcytotoxicity dye exclusion assay was performed to detect serum cytotoxic antibodies. However, instead of peripheral blood leukocytes (19), _SM_MSCs from the same donor were used as targets. Briefly, stem cells were tested in duplicate against neat and diluted sera (1:2 and 1:16 dilutions in PBS), incubating 1 µL of _SM_MSC and 1 µL of neat or diluted sera for 30 min at room temperature under oil. After that, 5 µL of serum rabbit complement were added and the samples were incubated for 1 h. All wells were dyed with 2 µL of 5% eosin and fixed with 5 µL of 10% formalin (pH 7.4). Plates were read in bright field microscope and results were graded according to the scoring system described by Berglund and Schnabel (19), where cell death < 10% is graded as 1; from 10%–19% as 2; from 20%–49% as 4; from 50%–80% as 6; and from 81%–100% as 8. Scores lower than 2 were classified as absence or non-significant antibody presence, whereas scores higher than 4 (6 and 8) were indicative of significant antibody production. Antibody titer was determined by observing the most elevated serum dilution capable of killing at least 80% of the donor _SM_MSC.

### 2.8 Statistical Analysis

Data were analyzed by t wo-way repeated measures ANOVA for time comparisons and Tukey’s test for multiple pairwise comparison. Statistical significance was considered at a level of 5% significance (P<0.05). All analyses were performed using GraphPad Prism 7.0 software.

## 3 Results

### 3.1 Characterization of _SM_MSC

**Figure 2:**
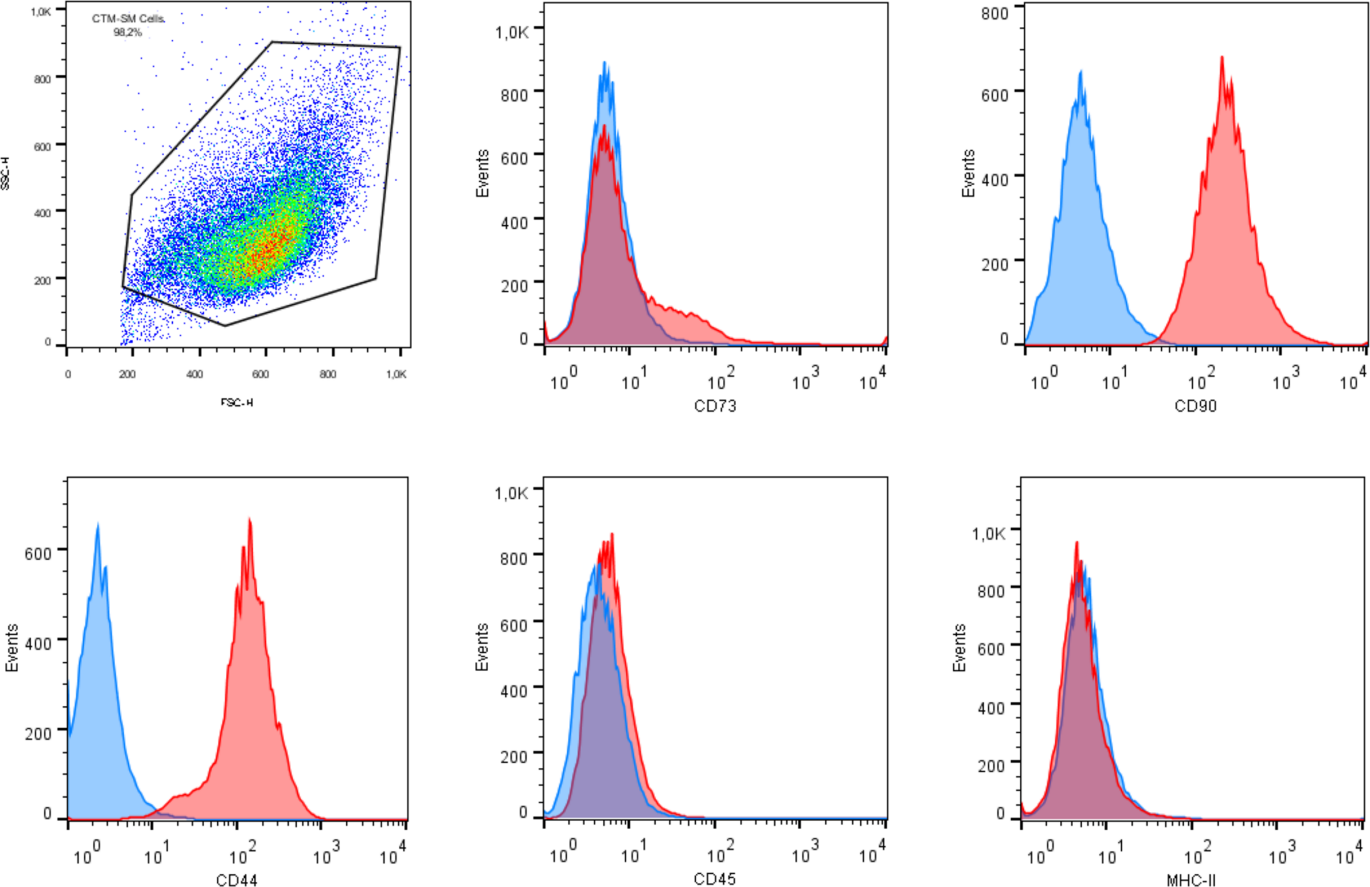
Flow cytometry analysis of mesenchymal stem cell surface markers in synovial membrane derived MSCs revealing expression of CD90, CD44, and CD73, and lack of expression of CD45 and MHC class II.

**Figure 3:**
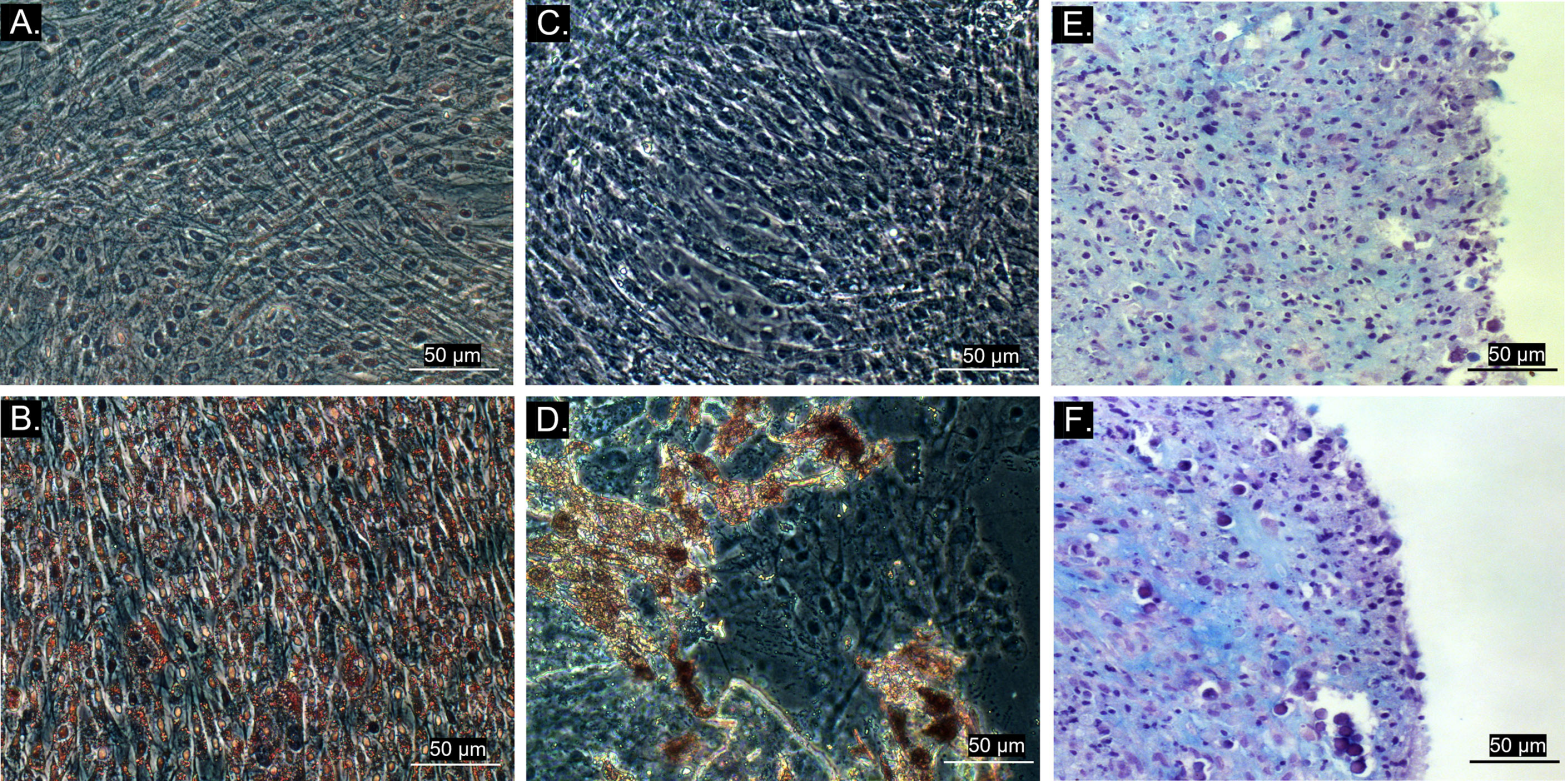
Bright field microscopy (20x objective) of MSC trilineage differentiation. **(A)** Control group of adipogenic differentiation; **(B)** Differentiated cell culture stained with Oil Red O, showing intracellular lipid droplets in red; **(C)** Control group of osteogenic differentiation; **(D)** Differentiated cell culture stained with Alizarin Red S, exhibiting extracellular hydroxyapatite crystals stained in red; **(E)** Control group of chondrogenic differentiation; **(F)** Differentiated tridimensional cell culture stained with Alcian Blue after 21 days, revealing chondral matrix sulphated proteoglycans stained in blue.

All cultures presented adherent spindle-shaped cells capable of clonal expansion. Flow cytometry revealed positive expression of CD90, CD73, and CD44 and absence of expression of CD45 and MHC class II (**Figures 1**, **2**). Differentiation was confirmed by bright field microscopy after staining. Adipogenesis was confirmed by the observation of intracellular lipid droplets stained with Oil Red O, whereas osteogenic differentiation was confirmed through the deposition of extracellular hydroxyapatite crystals stained with Alizarin Red S. Alcian Blue staining of chondrogenic differentiation highlighted extracellular chondral matrix deposition (**Figure 3**).

### 3.2 Clinical and Ultrasonographic Evaluation

Physical parameters remained within physiological range. Animals did not present abnormal physical exam findings such as tachycardia, tachypnea, abnormal intestinal motility, or hyperthermia. However, animals of the ALLO LPS group presented synovial effusion and joint sensitivity at the same time points after the first (T0 and T1) and the second _SM_MSC injection (T28 and T29). The groups ALLO and AUTO presented mild sensibility and joint effusion. Lameness scores of all groups reached grade 3/5 24h after each injection, decreasing until return to normality (absence of lameness) 14 days after each injection (T14 and T42, respectively). **Table 1** illustrates the clinical scores of synovial effusion and lameness.

**Table 1:** Clinical scores of synovial effusion and lameness grade.

ANIMAL	GROUP	PARAMETER	TIMEPOINT												
			T0	T1	T3	T7	T14	T21	T28	T29	T31	T35	T42	T49	T56
A1	AUTO	Synovial Effusion	-	++	++	+	+	+	-	++	++	+	+	-	-
		Lameness Score	-	II	II	II	I	-	-	III	I	I	-	-	-
A2	AUTO	Synovial Effusion	-	++	++	+	+	-	-	+	++	++	+	+	+
		Lameness Score	-	II	I	I	-	-	-	III	II	-	-	-	-
A3	AUTO	Synovial Effusion	-	++	++	++	+	-	-	++	++	+	+	+	+
		Lameness Score	-	II	II	I	-	-	-	III	II	I	I	-	-
A1	ALLO	Synovial Effusion	-	+++	++	++	+	-	-	+++	++	+	+	+	-
		Lameness Score	-	III	II	I	I	-	-	III	II	II	I	I	-
A2	ALLO	Synovial Effusion	-	++	++	+	+	+	+	++	+	+	+	+	+
		Lameness Score	-	II	II	I	-	-	-	III	II	II	I	-	-
A3	ALLO	Synovial Effusion	-	++	++	+	+	-	-	++	++	+	+	+	-
		Lameness Score	-	II	I	-	-	-	-	III	I	I	I	-	-
A4	ALLO	Synovial Effusion	-	++	++	+	-	-	-	++	++	+	+	-	-
		Lameness Score	-	III	II	I	-	-	-	III	I	I	I	-	-
A5	ALLO	Synovial Effusion	-	++	++	+	+	-	-	++	++	+	+	+	-
		Lameness Score	-	II	I	-	-	-	-	III	I	I	I	-	-
A6	ALLO	Synovial Effusion	-	+++	++	++	+	-	-	++	++	++	+	+	+
		Lameness Score	-	III	II	I	I	-	-	III	II	II	I	-	-
A1	ALLO LPS	Synovial Effusion	-	++	+	+	+	+	-	+++	++	++	++	+	-
		Lameness Score	-	II	II	I	I	-	-	III	III	II	I	-	-
A2	ALLO LPS	Synovial Effusion	-	++	++	+	+	+	-	+++	++	++	+	+	+
		Lameness Score	-	III	II	I	-	-	-	II	II	I	I	-	-
A3	ALLO LPS	Synovial Effusion	-	++	+	+	+	+	+	+++	++	++	+	+	+
		Lameness Score	-	III	II	II	I	-	-	III	II	II	I	-	-
A4	ALLO LPS	Synovial Effusion	-	++	++	+	+	-	-	+++	+++	++	+	+	+
		Lameness Score	-	III	II	I	I	-	-	III	II	II	I	-	-
A5	ALLO LPS	Synovial Effusion	-	++	++	+	+	+	-	++	++	+	+	+	-
		Lameness Score	-	III	II	I	-	-	-	III	II	II	I	-	-
A6	ALLO LPS	Synovial Effusion	-	+++	++	++	+	-	-	+++	++	++	+	+	+
		Lameness Score	-	III	II	II	I	-	-	III	II	II	I	-	-

Synovial effusion was classified as absent (-), mild (+), moderate (++) and severe (+++), whereas lameness was graded according to the AAEP scale, ranging from 0 to 5.

**Figure 4:**
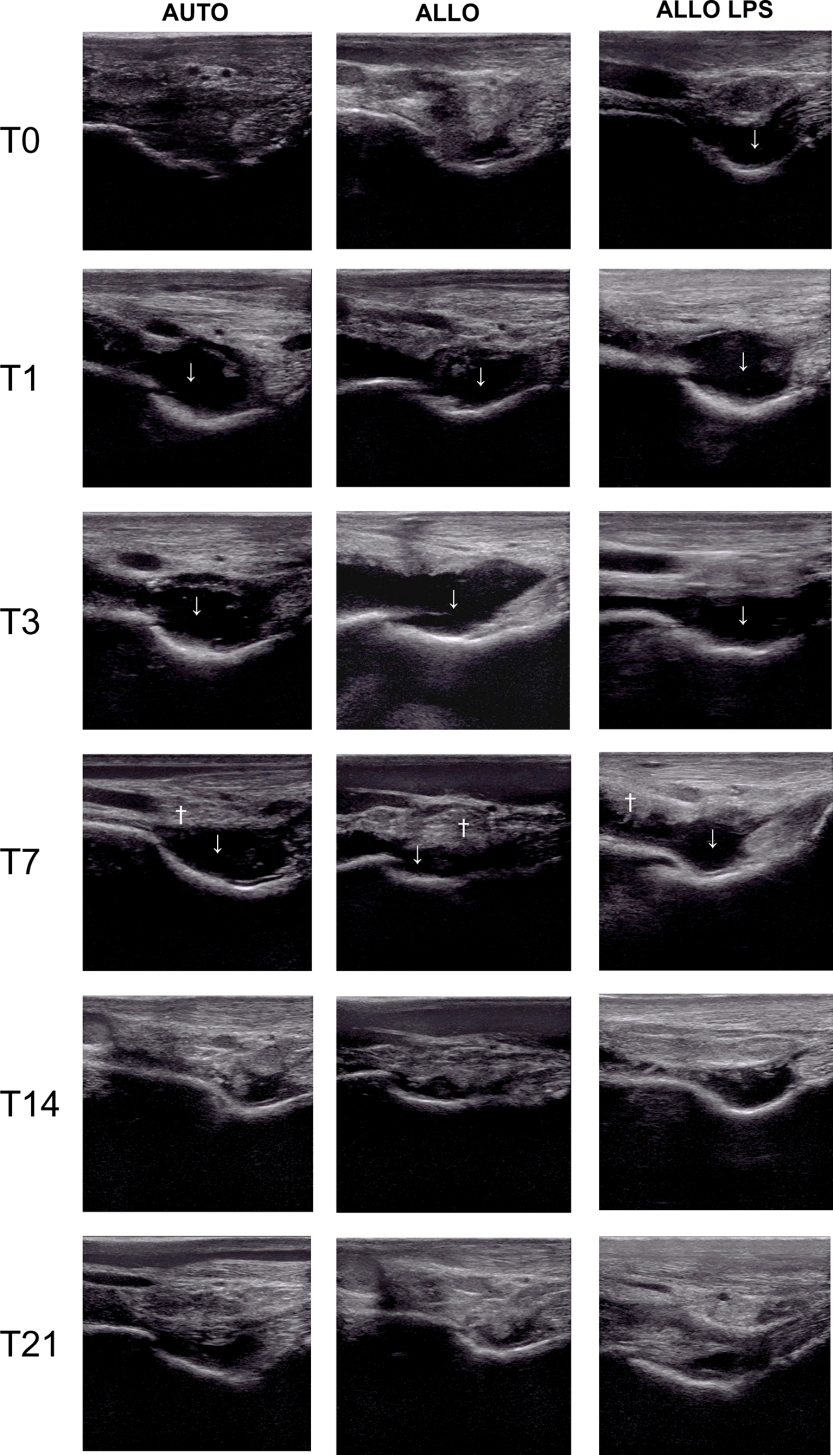
Ultrasonography of tibiotarsal joints before and after the first MSC injection, showing synovial effusion (↓) and synovial membrane hyperplasia (†). Synovial effusion was observed at T0 in ALLO LPS, and at T1 in AUTO and ALLO, and decreased at T14 in all groups. Synovial membrane proliferation occurred at T7 in all groups, although it was more evident in ALLO and ALLO LPS.

Ultrasonographic findings of synovitis were observed in all groups, whether being previously inflamed or not. Joint effusion occurred in AUTO and ALLO groups as soon as 24h after the first injection, decreasing after T14. Synovial membrane hyperplasia/proliferation was observed at T7 in all groups, being more apparent in ALLO and ALLO LPS (**Figure 4**).

**Figure 5:**
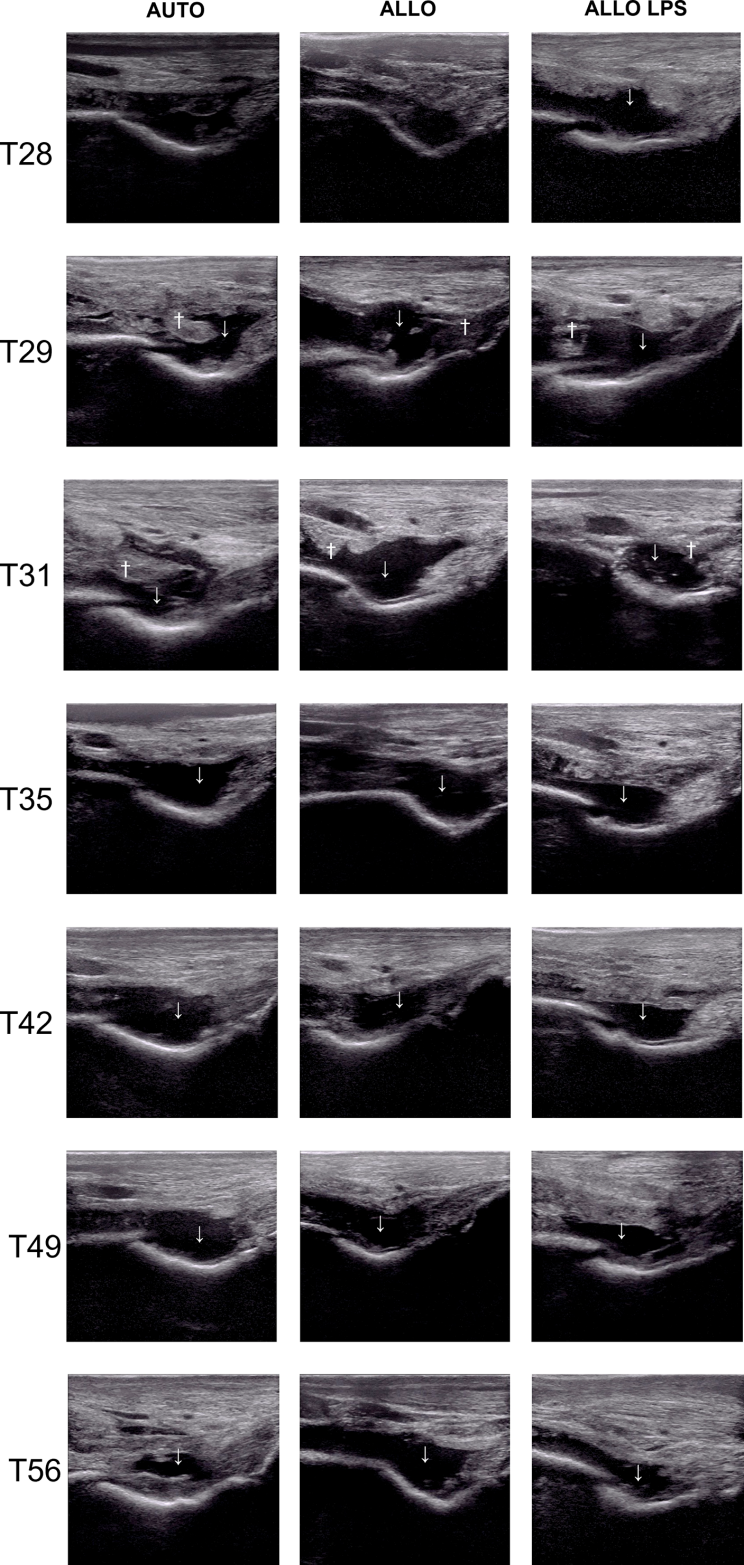
Ultrasonographic aspect of tibiotarsal joints before and after the second MSC injection. Extended synovial effusion (↓) and synovial membrane hyperplasia (†) can be noticed in all groups.

**Figure 6:**
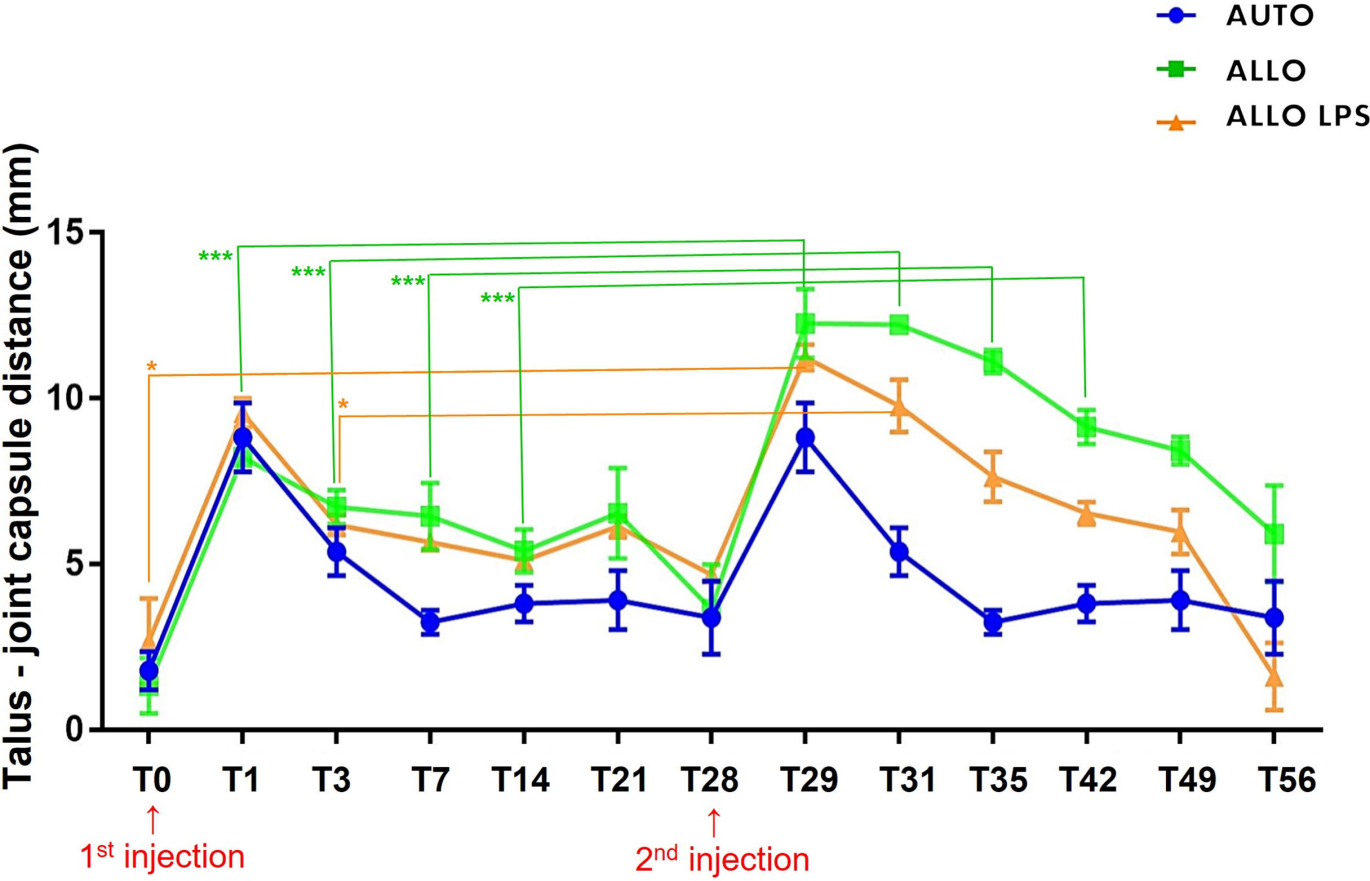
Mean ± SD values of synovial effusion, measured by the distance between the talus and the joint capsule. ALLO group presented statistical differences between first and second injection at T0, T1, T3, T7 and T14 (***P<0.005), whereas ALLO LPS group showed differences between first and second injection comparing T0 to T28 and T3 to T31 (*P<0.05).

A longer synovial effusion was observed after the second injection, remaining until T49 in the ALLO LPS group, and up to T56 in the ALLO group (**Figure 5**). A significant increase of tibiotarsal effusion (P<0.005) was observed in the ALLO group after the second injection comparing equivalent time points of both injections: T1 to T29, T3 to T31, T7 to T35, and T14 to T42, whereas a shorter increase in joint effusion occurred in the ALLO LPS group comparing T0 toT28 and T3 to T31 (P<0.05) (**Figure 6**).

### 3.3 Synovial Fluid Analysis

#### 3.3.1 Total Protein

**Figure 7:**
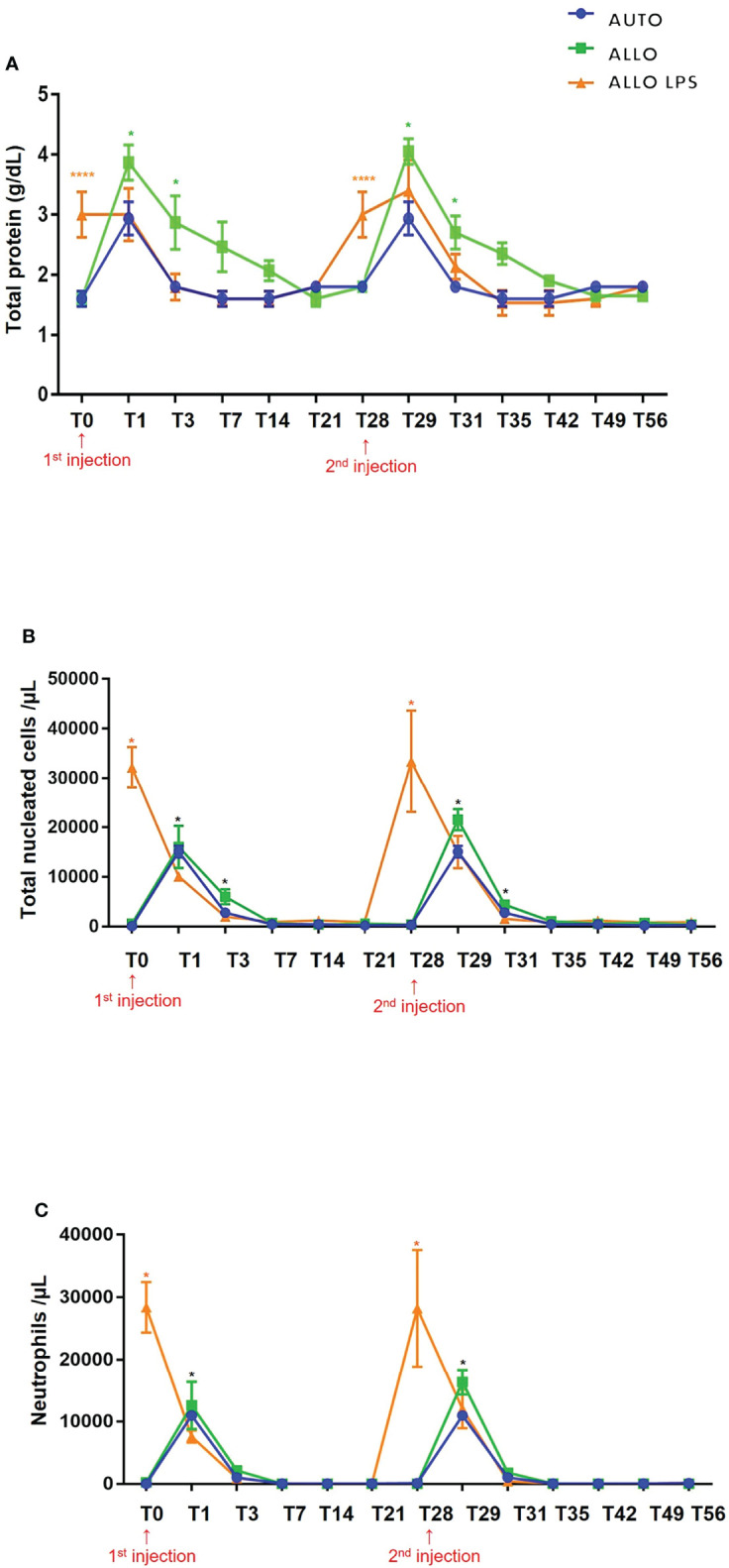
**(A)** Mean ± SD values of synovial fluid total protein (TP). Higher TP values were observed in ALLO LPS group at T0 and T28 (****P<0.0001). Statistical differences were observed between ALLO and AUTO groups at T1, T3, T29 and T31 (*P<0.05). **(B)** Mean ± SD values of synovial fluid total nucleated cell count (TNCC), showing absence of statistical differences between groups in first and second injections. Higher TNCC was observed in ALLO LPS group at T0 and T28 (*). Compared to the initial time point (T0 or T28), there was a significant increase in TNCC of all groups 1 and 3 days after _SM_MSC injection (T1 and T3; T29 and T31, respectively) (*P<0.05). **(C)** Mean ± SD values of synovial fluid neutrophil count, showing a higher neutrophil count in ALLO LPS group at T0 and T28 (*P<0.05), whereas in ALLO and AUTO the peak occurred at T1 and T29(*).

Overall, total protein (TP) levels peaked 24 h after the injections and decreased gradually to basal levels (< 2 g/dL) after 4 days. Although there was a transient increase in synovial fluid TP in all groups, no differences were observed between first and second _SM_MSC injection. Comparison of time points between all groups demonstrated higher TP in the ALLO LPS group at T0 and T28 (P<0.0001). Higher TP levels were also observed in ALLO group compared to AUTO group at T1, T3, T29 and T31 (P<0.05) (**Figure 7A**), indicating a lower decrease rate in TP of ALLO group.

#### 3.3.2 Total Nucleated Cell Count

Despite a moderate increase of SF TNCC in all groups, no differences were observed between first and second injection. Compared to the initial time point, there was a significant increase in SF nucleated cells of all groups 1 and 3 days after each injection (T1 and T3; T29 and T31, respectively) (P<0.05). Comparison between time points of different groups demonstrated higher TNCC in ALLO LPS group at T0 and T28 (P<0.05) (**Figure 7B**). All groups showed TP decrease up to 7 days after injections.

#### 3.3.3 Neutrophils

Compared to the initial time point, neutrophil count peaked 24 h after both injections (T1 and T29) in the AUTO and ALLO groups. However, no differences were observed between the first and second injections. Neutrophil count decreased to normal reference values from T3 on in all groups (**Figure 7C**).

### 3.4 Microcytotoxicity Assay

**Figure 8:**
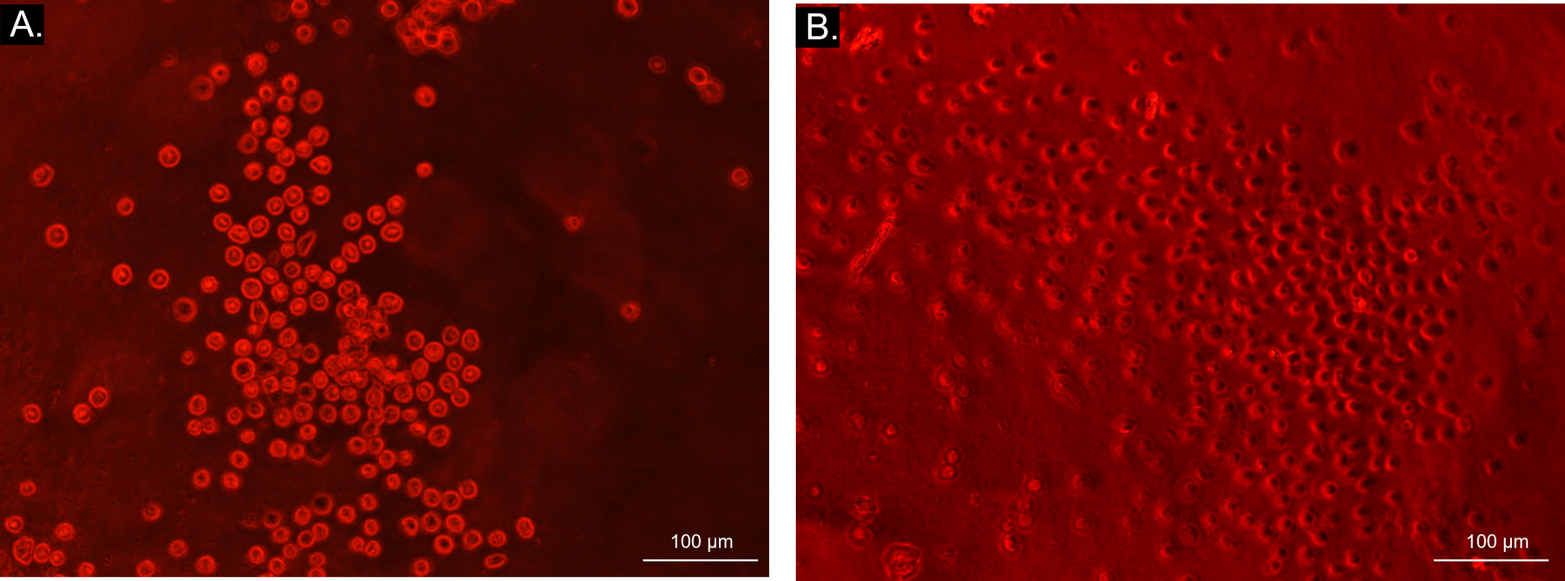
Bright field microscopy (10x objective) visualization of microcytotoxicity assay. Bright light and round cells represent live cells in AUTO group **(A)**, while dark-centered flat cells correspond to dead cells **(B)** in ALLO LPS group.

**Figure 9:**
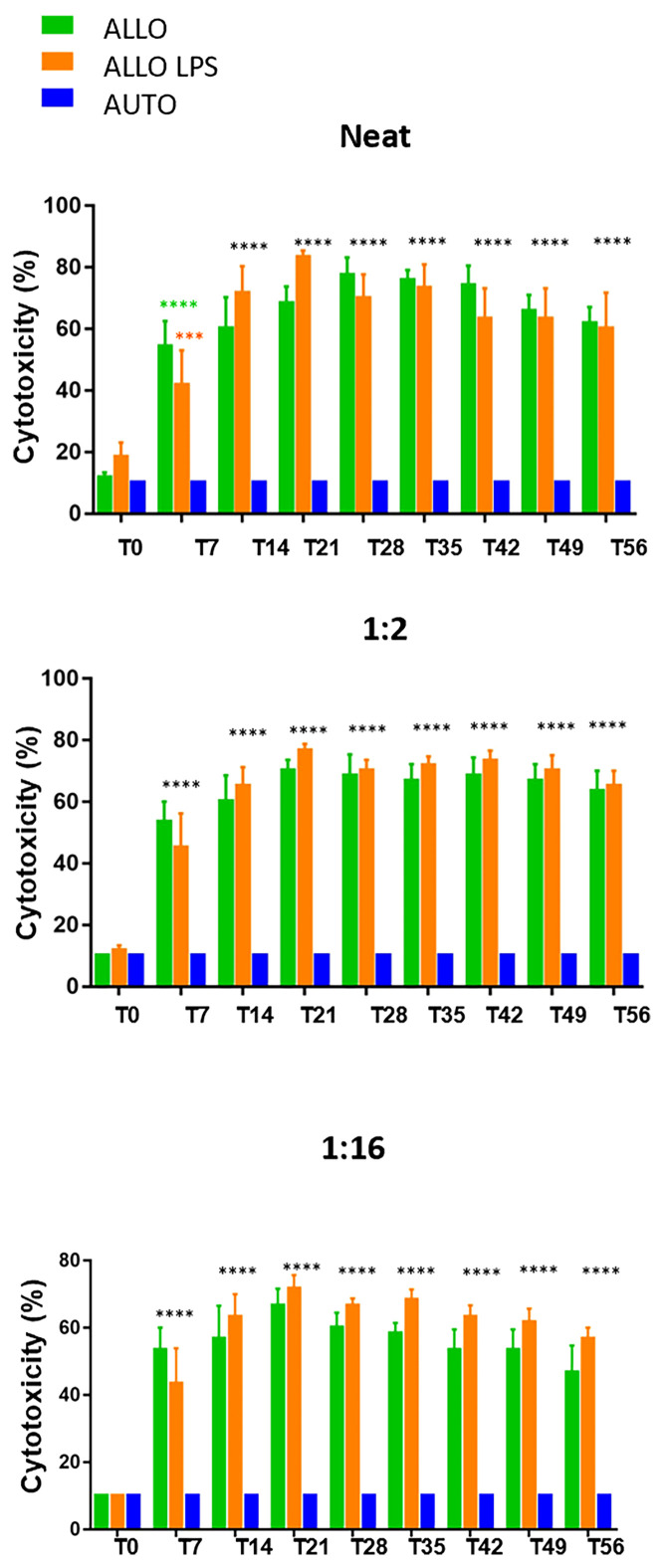
Microcytotoxicity assay before and after synovial membrane mesenchymal stem cell injections in neat, 1:2 and 1:16 sera dilutions. Initial time point did not reveal antibody production. However, comparison between T0 and T7 revealed P<0.0001(****) in neat serum of ALLO group and P=0.0038 (***) in ALLO LPS group. Subsequent time points showed significant antibody production in ALLO and ALLO LPS groups, from T14 to T56 (****P<0.0001) in all sera dilutions. No differences were observed between ALLO and ALLO LPS groups.

Sera dilution and neat sera showed consistent results between significant and non-significant antibody production, as **Figure 8** illustrates. Autologous sera and _SM_MSC incubation (AUTO group) did not lead to cell death at any time point or any serum dilution, confirming the absence of antibody production when autologous _SM_MSC was injected into healthy joints. Incubation of _SM_MSC and sera of initial time point (T0) also did not cause significant cell death (less than 10%) in the ALLO group. Although the ALLO LPS group also presented scores below the threshold of antibody production significance, two animals presented score 4 at T0. **Figure 9** illustrates cytotoxicity scores of different groups and time points.

At T7, serum of the ALLO and ALLO LPS groups presented significant antibody production in 50% (3/6) of animals. However, the animals that remained below the threshold of antibody production in the ALLO LPS group (3/6) presented lower scores at this time point. Comparison between T0 and T7 showed P>0.0001 in the ALLO group and P=0.0038 in the ALLO LPS group.

Fourteen days after first injection (T14), 67% (4/6) of the animals of the ALLO group presented antibody production, versus 83% (5/6) in the ALLO LPS group. Antibody production of the ALLO group rose to 83% (5/6) at T21 and T28, whereas 100% (6/6) of the ALLO LPS group produced antibodies against donor _SM_MSC at the same time points.

Seven days after the second _SM_MSC injection (T35), 100% (6/6) of the animals in the ALLO and ALLO LPS groups presented significant antibody production (score 6 or 8). After 14 days (T42) antibody production decreased to 83% in the ALLO group and remained 100% in the ALLO LPS group. At T49, 21 days after second injection, there was 67% of antibody production in the ALLO group compared to 83% in the ALLO LPS group. The last time point (T56) showed significant antibody production in 67% of both the ALLO and ALLO LPS groups.

Statistical analysis revealed no differences between the ALLO and ALLO LPS groups at any time point or any serum dilution (P>0.05), both differing from the AUTO group (P<0.0001) (**Figure 9**). Comparison between T0 and all subsequent time points revealed significant differences in the ALLO and ALLO LPS groups (P<0.0001 in all comparisons, except in the T7-T0 comparison of the ALLO LPS group, in which P-value was =0.0038).

## 4 Discussion

This study compared the inflammatory response and the cytotoxic antibody production after single and repeated intra-articular injections of allogeneic _SM_MSC in naïve healthy and experimentally inflamed joints. Our results demonstrate a significant increase in nucleated cells of the synovial fluid 24 h after first and second _SM_MSC injections, followed by the presence of cytotoxic alloantibodies in inflamed and healthy joints after first _SM_MSC injection. The second allogeneic injection led to a longer synovial effusion compared to autologous or first injections.

Previous studies have compared general immune response after intra-articular injections of MSCs in healthy (9, 11) and inflamed joints (25), describing mild and transient synovial effusion and lameness as consequences of injection. Results after the first _SM_MSC injection of our study corroborate the previous findings, since joint effusion was observed in all groups, including the AUTO group. However, even though after the second injection, the ALLO and ALLO LPS groups presented ultrasonographic synovitis findings (synovial proliferation) that remained until the last US evaluation. Differences in synovial effusion between first and second injections lasted longer in the ALLO group, indicating that the isolated administration of allogeneic _SM_MSC is also capable of evoking articular inflammation. These findings suggest that repeated allogeneic _SM_MSC injections can sensitize the recipient and, although might not cause intense inflammatory reactions, they can lead to mild yet lasting reactions potentially detrimental to MSC activity and viability, especially in further injections.

Even though specific mechanisms of articular inflammation after MSC injection are yet to be clarified (11), some authors suggest that inflammatory response can be originated by the introduction of proinflammatory cytokines from cell cultures (26) or xenogeneic compounds of the culture medium (27, 28). Considering that, conventional media was removed 48h before injection in our study. Still, since there was not a confirmation of the complete depletion of xenogeneic compounds from the media, the possibility of a transient inflammatory or immune reaction against reminiscent xenogeneic molecules needs to be considered. Another limiting factor of this study was the lack of equine leukocyte antigen (ELA) haplotype determination, which would have been a key analysis to identify mismatched, half-matched, or fully matched animals. Also, no peripheral blood leukocyte control group was utilized in the microcytoxicity assays, which could have been used to estimate the matching degree between donors and recipients, helping to discuss the results of antibody production and to support subsequent conclusions.

While often cited as immunoprivileged (29) and immune evasive (3), some authors show that MSCs attenuate the immune system but do not evade immune recognition whether in inflammatory or non-inflammatory environments. In fact, allogeneic MSCs are capable of eliciting antibody production and even presenting an MHC class II negative immunophenotypic profile (7). *In vivo* alloantibody production was described by Berglund and Schnabel (19) after intradermal injection of MSC in healthy joints, while Barrachina (15) demonstrated alloantibody formation in osteoarthritic joints that received allogeneic MSCs pre-exposed or not to inflammatory cytokines. Although both are inflammatory scenarios, our results showed higher alloantibody production after the second injection, leading to the inference that acute and chronic inflammation may play distinct roles in humoral response.

Given the absence of pre-existing antibodies verified at the initial time point of microcytotoxicity assays, our results showed specific alloantibody production leading to MSC opsonization and complement-dependent cytotoxicity, which corroborates previous studies regarding the existence of a specific immune response against MSCs (5, 19, 30). Inflammatory cytokines such as IFN-γ upregulate MHC II expression in equine MSC (6). Consequently, more expressed MHC II could elicit an early immune recognition and consequent MSC inactivation *via* both humoral and cellular mechanisms (15, 30–32).

As MHC II expression in MSC was expected to be increased in the ALLO LPS group due to the inflammatory process, higher alloantibody production was also expected in this group. Interestingly, the results of the present study demonstrate absence of differences in nucleated cell count, neutrophils, and antibody production after injection of _SM_MSC in healthy or previously inflamed joints of naïve horses. The absence of statistical differences between antibody production in inflamed and healthy joints indicates that even though inflammation is capable of increasing MSC immunogenicity, apparently it does not play a critical role nor interfere directly in antibody production on the first recipient exposure to allogeneic _SM_MSC. Although the study of Rowland et al. (30) showed that MHC mismatched MSCs are recognized by the immune system and elicit an immune response, leading to decreased MSC effects, the fact that animals produced antibodies as soon as 7 days after first injection, even being naïve for MSCs transplantation and without concomitant inflammation, indicates either half-matching or mismatching haplotypes, and may also postulate the existence of different surface molecules, other than MHC II, capable of eliciting specific immune response and alloantibody production.

Even under normal conditions, MHC class I is highly expressed by MSCs. MSCs expressing MHC-I, but not expressing MHC-II, and does not elicit immune responses in mixed leukocyte reactions *in vitro*, whereas MSCs expressing both MHC class I and II elicited CD4+ T-cell response (6). The diversity of cell types in an *in vivo* scenario promotes a broader and more complex immune reaction, including a CD8+ T-cell response and indirect allorecognition, which could recognize and produce antibodies against MHC class I (19). Unfortunately, our study did not include MHC class I in flow cytometric characterization of MSCs, so we cannot affirm whether MHC class I was expressed and consequently targeted by cytotoxic antibodies. However, allorecognition of MHC class I must also be considered as a potential cause for cell rejection. Nevertheless, the specific MSC target(s) for cytotoxic alloantibodies is (are) yet to be completely elucidated.

Higher scores of microcytotoxicity were observed after intradermal injection of bone marrow MSCs in horses (19). Some possibilities are highlighted after comparing both studies. First, the route of administration can affect the immune response. According to Mohanan (33), intradermal injections of antigens generate higher IgG2a levels than intramuscular or subcutaneous routes. Also, skin is densely populated by immune-related cells such as keratinocytes capable of secreting immunostimulatory cytokines (34) and phagocytes that present antigens in secondary lymphoid organs and initiate an immune response of great magnitude (35). Second, horses of the study of Berglund and Schnabel (19) had ELA haplotypes determined and were known to be mismatched, whereas the exact matching conditions between the horses of our study are unknown. Horses also received 3 to 5 times more MSCs compared to our study, and the source of MSC was different. While the influence of MSC source on immune response remains unclear, it is important to point out that different experimental conditions may lead to different results.

Overall, data analyses and comparisons with similar studies indicate that single and repeated intra-articular allogeneic MSCs injections can trigger cellular and humoral immune responses in the recipient, with production of cytotoxic alloantibodies that might partially or totally impair MSCs activity. However, some disorders require prompt intervention and thus allogeneic injections are indicated due to the easier and faster isolation and expansion. In cases where a second or subsequent MSC injection is needed and the ELA compatibility is not determined, we propose that the use of autologous MSCs can be beneficial. Once the tissue selected to be the source of MSC is harvested on the day of the first injection, autologous MSCs isolation can be performed in the meantime between first and second injections. This way, the risk of immune allo-reactions is eliminated and MSCs can exert their full therapeutic properties.

## 5 Conclusion

This study demonstrated that specific cytotoxic alloantibody production can occur as early as 7 days after first MSC injection, becoming worse in subsequent injections, whether the recipient tissue is inflamed or not. These findings shed light on the importance of comprehension of inflammatory and immune responses related to *in vivo* MSC injections. Until then, strategies aiming to prevent or reduce MSC immunogenicity and enhance MSC biological properties must be developed and applied, since the synovial environment presents a distinct inner barrier layer with special immunological mechanisms. Special attention is required in allogeneic cell-based therapies, particularly in repeated injections. The use of autologous MSC injections is indicated to avoid allo-reactions and it might be considered in cases where subsequent MSC injections are required.

## Data Availability Statement

The raw data supporting the conclusions of this article will be made available by the authors, without undue reservation.

## Ethics Statement

The animal study was reviewed and approved by Ethics Committee of São Paulo State University (0240/17).

## Author Contributions

Conceptualization: GR, AK, and AA; Data curation: GR and AK; Formal analysis: GR, AK, EP, MR, FS, RB, AB, and AA; Funding acquisition: AA; Investigation: GR, AK, EP, FS, RB, JP, MR, AB, and MG; Methodology: GR, AK, FS, JP, and AA; Project administration: AA; Resources: MG and AA; Supervision: AA; Visualization: GR and AK; Writing – original draft: GR and AK; Writing – review and editing: GR, AK, and FS. All authors contributed to the article and approved the submitted version.

## Funding

We thank the São Paulo Research Foundation (FAPESP grant number 2017/14460–4 and 2017/12815-0), the Coordination for the Improvement of Higher Education Personnel (CAPES funding code 001) and the National Council for Scientific and Technological Development (CNPq grant number 155915/2019-3 GD), for funding this research project.

## Conflict of Interest

The authors declare that the research was conducted in the absence of any commercial or financial relationships that could be construed as a potential conflict of interest.

## Publisher’s Note

All claims expressed in this article are solely those of the authors and do not necessarily represent those of their affiliated organizations, or those of the publisher, the editors and the reviewers. Any product that may be evaluated in this article, or claim that may be made by its manufacturer, is not guaranteed or endorsed by the publisher.
